# The Effect of Lentivirus-Mediated PSPN Genetic Engineering Bone Marrow Mesenchymal Stem Cells on Parkinson’s Disease Rat Model

**DOI:** 10.1371/journal.pone.0105118

**Published:** 2014-08-13

**Authors:** Xiaofeng Yin, Huamin Xu, Yunxia Jiang, Wenshuai Deng, Zeyu Wu, Hengwei Xiang, Peng Sun, Junxia Xie

**Affiliations:** 1 Department of Neurosurgery, Affiliated Hospital of Medical College, Qingdao University, Qingdao, China; 2 Department of Physiology, Shandong Provincial Key Laboratory of Pathogenesis and Prevention of Neurological Disorders and State Key Disciplines: Physiology, Medical College of Qingdao University, Qingdao, China; 3 Nursing College of Qingdao University, Qingdao, China; University of Freiburg, Germany

## Abstract

Persephin (PSPN) is one of the neurotrophic factors of the glial cell line-derived neurotrophic factor (GDNF) family ligands (GFLs) which have been found to promote the survival of specific populations of neurons. The aim of this study was to assess the potential therapeutic function of gene-modified mesenchymal stem cells (MSCs)-Lv-PSPN-MSCs in 6-OHDA-induced Parkinson’s disease (PD) rats models. Here, we worked on the isolation, purification, identification and amplification of MSCs in vitro. The expression analysis revealed that several of the neural marker proteins like nestin, GFAP and S100 were expressed by rat MSCs. MES23.5 cells co-cultured with Lv-PSPN-MSCs showed less 6-OHDA induced cell death than control cells in vitro. When Lv-PSPN-MSCs were injected into the striatum of PD rats, we observed the survival rate, migration, differentiation and the behavior change of PD rats. We found that Lv-PSPN-MSCs showed higher survival rate in rat brain compared with Lv-null-MSCs. Rotational behavior showed that rats receiving Lv-PSPN-MSCs showed the most significant improvement compared with those in other groups. HPLC results showed the content of DA in striatum of rats which received Lv-PSPN-MSCs was highest compared with those in other groups. In conclusion, our results suggest that transplantation of Lv-PSPN-MSCs can lead to remarkable therapeutic effects in PD rats.

## Introduction

Parkinson’s disease (PD) is the second most common neurodegenerative disease in the world. It is characterized by the progressive degeneration of dopaminergic neurons in locus ceruleus and substantia nigra (SN), which leads to the reduction of dopamine (DA) levels in striatum [1]. The progressive loss of nigral dopaminergic neurons results in a series of clinical symptoms, including hypokinesia, static tremor, muscle rigidity, and abnormal posture and pace [2]. Although drug therapy and surgical treatment can relieve the symptoms, they cannot prevent the progression of the disease and fundamentally cure PD, because the loss of neurons cannot be restored. Cell transplantation is a therapy to replace the loss of neurons in SN, the efficacy is apparent but not lasting. Therefore, the present study focuses on how to develop more effectively long-term therapies for PD.

Mesenchymal stem cells (MSCs) are from the bone marrow stroma which have the advantage of easy isolation, self-renewal, multi-potential and low immunogenicity [3]. MSCs have been widely used in tissue engineering to regenerate different cells/tissues and repair damaged tissues after being transplanted into striatum of PD rats [4], [5]. It has also been reported that MSCs can differentiate into different cell types under appropriate cellular conditions in vitro, such as skeletal muscle cells [6], endothelial cells of the blood vessels [7] and hepatocytes [8]. In addition, the intravenous administration of MSCs into rat has been suggested to differentiate into neuron- and glial-like cells in the brain [9]. Despite so many reports on the bone marrow-derived mesenchymal stem cells differentiation capacity, there is still a problem about relatively lower survival rate of MSCs in vivo. Therefore, it is definitely indispensable to discover a better reliable method to improve MSCs differentiation rate and survival rate in vivo.

Neurotrophic factors can improve survival rate of nerve cells and can reduce the degeneration of neurons induced by trauma, diseases or neurotoxic chemicals [10]. These factors are distinctly significant to promote the proliferation, differentiation and survival of the nerve cells. The expression of these neurotrophic factors was improved during neuronal impairment [11]–[13]. Further study showed that these neurotrophic factors were endogenous protective agents that can be increased during neuronal impairment to protect the damaged nerve cells [14], indicating the important clinical significance of these neurotrophic factors for the treatment of PD.

Persephin (PSPN) is one of the neurotrophic factors of the GDNF family which currently consisting of GDNF, neurturin (NTN), PSPN and artemin/neublastin (ART). Previous studies have showed that PSPN is as potent and efficacious as GDNF at promoting the survival of midbrain dopamine neurons in vitro and could prevent the loss of dopamine neurons of mice receiving intrastriatal 6-OHDA injections [15]. However, there is little evidence to investigate the rescue effect of PSPN in PD in vivo. It has been reported that prenatal ventral mesencephalon and the newborn striatum express high levels of PSPN mRNA. However, PSPN mRNA levels were downregulated in the ventral mesencephalon and the striatum after birth. It was reported that signaling of PSPN is mediated by a receptor complex which is comprised of the glycosylphosphatidylinositol (GPI)–linked GDNF family GFRα4 receptor and transmembrane signal-transducing module receptor tyrosine kinase RET [16], [17]. Evidence also showed midbrain dopamine neurons expressed its preferred receptor GFRα4 [15], indicating the possible neurotrophic effect of PSPN on dopaminergic neurons via GFRα4 in PD.

Therefore, intrastriatal grafting of MSCs engineered to overexpress PSPN via recombinant lentivirus were used to investigate the neurotrophic effect of exogenous PSPN gene on 6-OHDA-induced PD rats. Our results showed PSPN increased survival rate of implanted MSCs, enhanced differentiation of MSCs to neuron- and glial-like cells, improved rotational behavior of PD rats and increased the content of DA in striatum. This provides evidence for the application prospect of MSCs expressing PSPN in neurodegenerative diseases.

## Materials and Methods

### Isolation and Cultivation of rMSCs

All experimental procedures were in accordance with the National Institute of Health Guide for Care and Use of Laboratory Animals and were approved by the Animal Ethics Committee of Qingdao University. Adult 8-week-old male SD rats were anesthetized with intraperitoneal (i.p.) injection of 0.5 g/kg chloral hydrate (RiedeldeHaen, Seelze, Germany). rMSCs were collected from femurs by flushing the shafts with α-modified Eagle’s medium (α-MEM; GIBCO, USA), serum free, using a syringe needle with the 10th. Cells were disaggregated by gentle pipetting several times. The suspension of cells obtained was centrifuged at 1000 rcf for 5 min. Remove the supernate and resuspend in a culture medium consisting of α-MEM with 10% fetal bovine serum (FBS; Hyclone, Logan, UT), 100 U/mL penicillin, 100 µg/mL streptomycin (Lonza, Verviers, Belgium). Cells were plated at a density of 3×10**^6^** cells/cm^2^ in 25 cm^2^ culture flasks (Falcon, Becton Dickinson).

rMSCs cultures were maintained at 37°C in a humidified atmosphere containing 5% CO_2_. After 48 h, the nonadherent cells were removed, and the cells attaching to the culture flasks were cultured in α-MEM with 10% (v/v) FBS, with a change of medium every 3–4 days. When rMSC cultures reached 80–90% confluence, cells were detached by using 0.05% (w/v) trypsin in 0.1% (w/v) EDTA (Lonza, Verviers, Belgium). The suspension of cells obtained was centrifuged at 1000 rcf for 5 min. Remove the supernate and resuspend in α-medium and the cells were used for further experiments.

### Determination of cell-surface antigen profiles

Cells were seeded into culture slide. When it reached 80–90% confluence, rMSCs were fixed with 4% paraformaldehyde for 40 minutes and then were washed with PBS. Fluorescinisothiocyate (FITC)-conjugated antibodies (1∶500, Sigma-Aldrich) against rat CD34 or CD44, and phycoerythrin (PE)-conjugated antibodies (1∶200, Invitrogen) against rat CD45 or CD90 (BD Pharmingen, San Diego, CA) were added to wells in the dark respectively. Then rMSCs were covered with cover glasses and observed under a fluorescent microscope (Zeiss, Oberkochen, Germany).

### Lentivirus-mediated Expression of PSPN in rMSCs

Recombinant lentivirus containing rat PSPN gene was provided by Shanghai GeneChem. The rMSCs were seeded in 12-well culture plates at a confluence of 50–60%, and then lentivirus at multiplicity of infection (MOI) of 10 was added to the medium. After 72 h of infection, green fluorescent protein (GFP) was observed under a fluorescence microscope (Zeiss, Oberkochen, Germany).

### Western Blots

After the infection with lentivirus (10 MOI) for 72 h, cells were washed with PBS, and whole cells extracts were prepared. A total of 15 ug of protein from each sample was separated using 10% SDS–polyacrylamide gels and transferred to PVDF membranes. After 2 h blocking with 10% non-fat milk at room temperature, the membranes were incubated with rabbit-anti-rat PSPN (1∶1000) antibodies over night at 4°C. Anti-rabbit secondary antibody conjugated to horseradish peroxidase was used at 1∶10000. Blots were also probed with anti-β-actin antibody (1∶8000) as a loading control. Cross-reactivity was visualized using ECL western blotting detection reagents and analyzed by scanning densitometry using a UVP Image System (Upland, CA, USA).

### Semiquantitative Reverse Transcription-PCR

rMSCs were pretreated with Lv-PSPN for 72 h, cells were lysed and total RNA was extracted from each sample using TRIzol reagent (Gibco Invitrogen Corp, Carlsbad, CA, USA). About 2 µg of total RNA were reversed transcribed in a 20-µl reaction using the AMV reverse transcription system (Promega Corporation, Madison, WI, USA). The sequences of the PCR primers for PSPN and the housekeeping gene glyceraldehyde-3-phosphate dehydrogenase (GAPDH) were 5′-ATAGTCGACATGGCCCTGTCTGGTCCATGCCAGC-3′(PSPN forward) and 5′-ATACTCGAGTCAGCCACCACAGCCGCAGGC-3′(PSPN reverse) and 5′- TGTTCCAGTATGATTCTACCCA-3′ (GAPDH forward) and 5′- GGTAGGAACACGGAAGGC-3′ (GAPDH reverse). PCR system using the following cycle parameters: 1 cycle of 95°C for 1 min, and 40 cycles of 95°C for 20 s, 65°C (PSPN), or 59°C (GAPDH) for 20 s and 72°C 18 s. The PCR products were electrophoresed on 1% agarose gel, stained with ethidium bromide, and ethidium bromide-stained gels were scanned and qualified using Tanon Image Software. The levels of PSPN mRNA were expressed as their respective ratios to GAPDH mRNA.

### Immunofluorescence staining of cells

The rMSCs infected with recombinant Lv-PSPN were fixed with 4% para­formaldehyde for 40 minutes, washed three times with PBS and blocked with 5% bovine serum albumin at room temperature for 1 h, followed by incubation with primary antibodies which were presented in Table 1 (1–7) at 4°C overnight. After washing three times, fluorochrome-conjugated secondary antibody (Invitrogen, Karlsruhe, Germany) was added to incubate cells at room temperature for 60 minutes. Hoechst 33258 (Sigma, USA) was used to stain nuclei. The presence of the proteins was examined under a fluorescence microscope (Zeiss, Oberkochen, Germany).

**Table 1:** List of primary antibodies used in this study.

Antibody	Source	Dilution
1 Anti-PSPN	Affinity Bioreagents	1∶1000
2 Anti-S100 mAb	Sigma	1∶1000
3 Anti-IBA-1mAb	Chemicon	1∶200
4 Anti-β3-tubulin	Covance, Richmond, CA, USA	1∶8000
5 Anti-NES mAb	Sigma	1∶2000
6 Anti-Nestin mAb	DSHB	1∶200
7 Anti-GFAP	Mannheim, Germany	1∶2000
8 connexin 43	Sigma	1∶500
9 Anti-Map-2 mAb	Sigma, Deisenhofen, Germany	1∶500

### The MTS Assay for Metabolic Activity

(3-(4,5-dimethylthiazol-2-yl)-5-(3-carboxymethoxyphenyl)-2-(4-sulfophenyl)-2H-tetrazolium) assay was used as an indirect colorimetric measurement of metabolic activity. Briefly, rMSCs cells were seeded in 96-well plates (1×10^4^ cells/well) in α-DMEM supplemented with 5% FBS and antibiotics. 12 h after plating, the cells were treated with vehicle, Lv-null (10 MOI) and Lv-PSPN (10 MOI) respectively for 72 h. The medium was discarded and replaced with 100 ul of MTS/PMS solution (Promega, Madison, WI, USA) according to the manufacturer’s instructions. After incubated at 37°Cfor 1 h, an absorbance at 490 nm was measured on amicroplate reader (Bio-Rad Laboratories Inc., CA, USA). Metabolic activity was expressed as a percentage of the control cells treated with vehicle and was designated as 100%.

### Flow Cytometric Analysis of Cell Apoptosis

Lv-PSPN-MSCs or Lv-null-MSCs were cocultured in an upper chamber with 6-OHDA exposed MES23.5 cells in a Transwell chamber. Apoptosis was measured 24 h later using flow cytometry. MES23.5 cells were then trypsinized and collected by centrifugation, and were treated with both 7-aminoactinomycin D (7-AAD) and annexin V-PE (Abcam, Cambridge, MA, USA) for 15 min. Fluorescence flow cytometric analyses of apoptosis were performed using a Guava EasyCyte Mini instrument (MILLIPORE Guava technologies, Billerica, MA, USA). Gating was adjusted using 7-AAD staining with dot plots displaying FL3-7-AAD on the y-axis and FL2 annexin V-PE on the x-axis. More than 1,0000 events were collected for each sample.

### Animals and Transplantation Surgery

All experimental procedures were in accordance with the National Institute of Health Guide for Care and Use of Laboratory Animals and were approved by the Animal Ethics Committee of Qingdao University. Male Sprague–Dawley rats (200–250 g) received unilateral stereotaxic injections of 16 µg 6-OHDA into the right medial forebrain bundle (MFB). Coordinates were set according to the atlas of Paxinos and Watson [18]. The injection rate was 1.0 µl/min, and the cannula was left in place for another 5 min before slowly retracting it. Rats reaching a level of at least 7 rotations/min were regarded as PD model rats.

All PD rats (n = 50) were divided into five groups. Group I (n = 10): PD rats that received nothing; group II (n = 10): PD rats that received vehicle only; group III (n = 10): PD rats that received MSCs; group IV (n = 10): PD rats that received Lv-null-MSCs; group V (n = 10): PD rats that received Lv-PSPN-MSCs.

Two weeks after lesion surgery, a total of 1×10^6^ cells/5 µl MSCs were injected into striatum ipsilateral to the unilateral 6-OHDA injection. PD rats were anesthetized by intraperitoneal (i.p.) injection of 0.5 g/kg chloral hydrate (RiedeldeHaen, Seelze, Germany), and placed in a stereotaxic frame. About 5 mm incision was made in the scalp 2 mm lateral to the bregma. A burr hole was made in the bone with a dental drill. The transplants were placed 4.8 mm lateral to the midline, 2.12 mm posterior to the bregma, and 6 mm inferior to the dura. Cell suspensions were slowly injected using a 5 ul Hamilton syringe over a 5-minute period. The needle was left in place for further 5 minutes before slowly extracting. After cell injection, the skin incision was sutured. Following rMSCs injection the animals received daily subcutaneous cyclosporin A injections (10 mg/kg).

### Immunofluorescence Staining of Tissue Sections (bleach section)

For the investigation of neural marker expression by implantable rMSCs, brain sections (30 mm) were prepared with slicing machine. Brain sections were washed in PBS and fixed in 4% formaldehyde. Immunocytochemistry was performed at room temperature. Brain sections were blocked with 2% goat serum for 30 min, treated with 0.025% Triton X-100 for 30 min and rinsed in PBS. The primary antibodies used are presented in Table 1 (5–8). Incubations were performed overnight at 4°C. After washing three times, fluorochrome-conjugated secondary antibody (Invitrogen, Karlsruhe, Germany) was added to wells at room temperature for 60 min. After antibody incubations nuclear DNA was stained with Hoechst 33258. Preparations were coverslipped with Entellan (Merck, Darmstadt, Germany). Analysis of mounted specimens was carried out using an Axiophot fluorescence microscope (Zeiss, Oberkochen, Germany).

### Rotational Behavioral Assessment

Rotational behaviours were tested each week with apomorphine (APO, 0.1 mg/kg, s.c.) in automated “rotometer” bowls for 30 min in different groups.

### Biochemical Examination after Transplantation

At 8 weeks after-transplantation, striatal concentrations of DA, DOPAC and HVA were measured by high-performance liquid chromatography (HPLC) with an electrochemical detector. Rats were decapitated at a temperature of 4°C under sterile conditions. The brain tissue was rinsed with ice-cold PBS, precisely weighed, and homogenized in an ice-cold perchloric acid and EDTA solution (0.4 mol/l HClO_4_, 0.5 mmol/l Na_2_-EDTA, 0.01% L-cysteine) to yield a 10% (w/v) homogenate at 0°C. The homogenates were centrifuged at 14,000 rcf at 4°C for 15 min. After centrifugation, the supernatants were removed, and 0.5 volume of potassium salt solution (20 mmol/l potassium citrate, 300 mmol/l KH_2_PO_4_, 2 mmol/l Na_2_-EDTA) was added at 0°Cfor 15 min. The precipitates were then centrifuged at 14,000 g at 0–4°C for 15 min. After centrifugation, the supernatants were frozen at −80°C or immediately applied to the HPLC system. Dialysis samples were assayed for DA, DOPAC and HVA by HPLC with electrochemical detection. The samples were placed in the 717 Plus Autosampler (Waters, Milford, MA, USA) connected to the 2465ECD (Waters, Milford, MA, USA) equipped with a C18 reverse-phase column (4.6×75 mm, 3.5 Um; Waters, Milford, MA, USA). The samples were eluted by mobile phase (100 mM Na-citrate, 0.1 mM EDTA, 75 mM Na_2_HPO_4_, 2 mM NaCl, 1 mM C-7 at pH 3.9, 10% methanol) at a flow rate of 0.6 ml/min. DA, DOPAC and HVA levels were calculated by extrapolating the peak area from a standard curve (ranging from 1 nM to 100 nM of mixed DA, DOPAC and HVA). The identification of peaks was carried out by comparison with standards. The amounts of DA in each sample were quantified by comparing the peak area of the samples to those of the standards.

### Statistical Analysis

Statistical analysis was conducted using GraphPad Prism software (GraphPad Software Inc., USA). The data are expressed as mean ± SD and analyzed by two-way ANOVA, t-test or one-way ANOVA. *P*<0.05 was considered to be statistically significant.

## Results

### Morphological Characteristics and Determination of rMSCs

**Figure 1:**
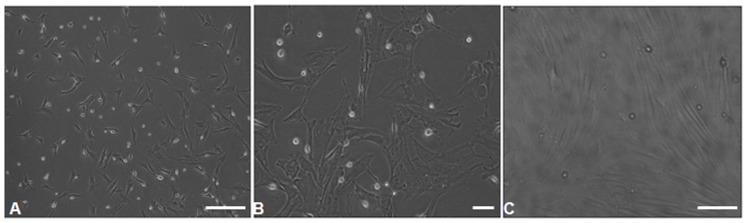
Morphological characteristics of cultured rMSCs. Cells were spindle-shaped **A** (×100), **B** (×200). The attached cells were forming typical spindle shape **C** (×100).

In order to characterize rMSCs, cells were isolated by their adherence to plastic dishes and grown for 4–5 passages in α-medium. rMSCs contained two morphologically distinct cell types: spindle-shaped cells and large, flat cells. Most cells, however, were spindle-shaped (Fig. 1 A–B). The attached cells were forming typical spindle shape under a micro­scope. (Fig. 1 C).

**Figure 2:**
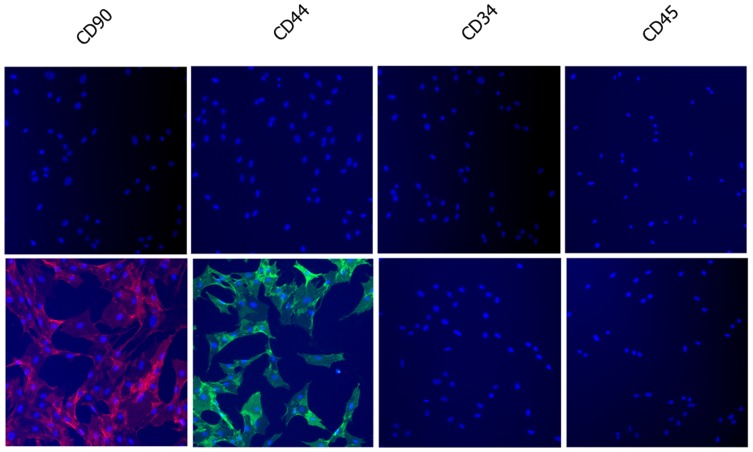
Determination of cell-surface antigen profiles of rMSCs using immunofluorescence. Cells were stained with isotype control antibodies (upper panels) and CD44, CD90, CD34 and CD45 antibodies (lower panels). Results showed cells were positive for CD44 and CD90 and negative for CD34 and CD45.

The surface-antigen profiles of the rMSCs were determined by staining with rat-specific monoclonal antibodies, followed immunofluorescence (Fig. 2). Most cells were positive for CD44 and CD90 but were negative for CD34 and CD45.

### Neural Marker Expression by rMSCs

**Figure 3:**
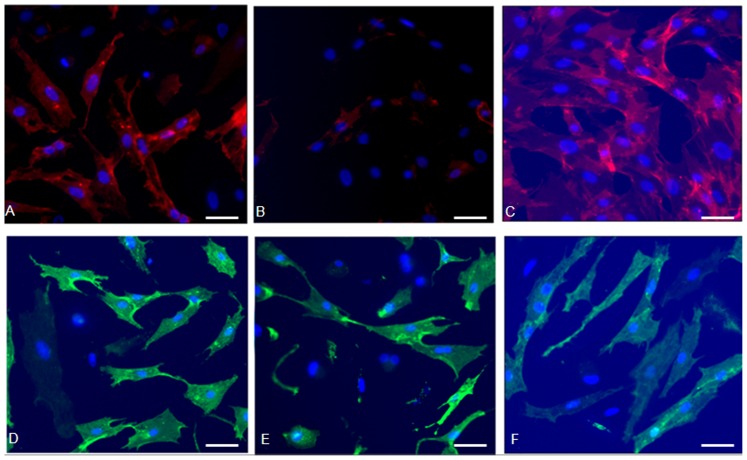
Neural marker expression in rMSCs cultured inα-medium. (**A**) Expression of the astrocyte marker GFAP (red) in 80±2.6% of rMSCs, the cells rarely exhibit neuronal-like morphologies. (**B**) The neuronal stem cell marker Nestin staining (red) in 10.8±5.9%of the rMSCs. (**C**) Expression of the neuronal markerβ3-tubulin (red) in 89.2±4.5 of the total rMSCs population. (**D**) Almost 65±2.2% of the rMSCs is immunopositive for the neuronal progenitor marker NES (green). (**E**) About 77.2±4.2% of the rMSCs expresses the glial cell marker S100 (green). (**F**) Also the microglia maker IBA-1 (green) is expressed by a majority of cells. Blue: Hoechst 33258 counterstaining. Scale bar = 200 µm.

Further experiments revealed that 80±2.6% of rMSCs were immunopositive for the astrocyt marker GFAP (Fig. 3 A). In accordance with a virtually general expression of GFAP, rMSCs were also positive for the neuronal stem cell marker Nestin (10.8±5.9%) (Fig. 3 B). Additionally, a virtually general expression of the neuronal marker β3-tubulin (89.2±4.5) could be detected (Fig. 3 C). Furthermore, 65±2.2% of rMSCs expressed the neuronal progenitor marker NES (Fig. 3 D). 77.2±4.2% rMSCs could express a number of glial cell markers S100 (Fig. 3 E). A subpopulation of 80.7±6.4% rMSCs was immunopositive for the microglia maker IBA-1 (Fig. 3 F).

### Expression of PSPN Genes in Transduced rMSCs

**Figure 4:**
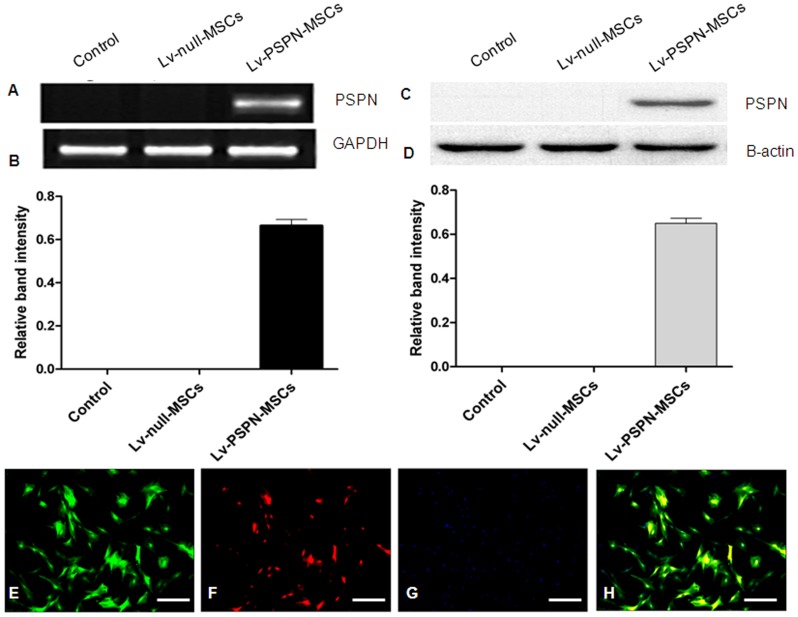
Expression of PSPN genes in transduced rMSCs detected by Western blots, RT-PCR and immunofluorescence. (**A**) RT-PCR detection of PSPN expression in rMSCs. (**B**) The relative density of bands was calculated using GAPDH as a loading control. (**C**) Western blots analysis of cell extracts from rMSCs after transduction with Lv-PSPN. Equal amounts of cell extracts were used in all lanes. β-actin was used as a loading control. (**D**) Western blotting films from three independent experiments were scanned and the relative density of protein bands was calculated using β-actin as a loading control. At least three independent experiments were performed. Data are presented as means±SD. Immunofluorescence staining for PSPN (E–H). (**E**) EGFP-labeled rMSCs. (**F**) Expression of PSPN genes in transduced rMSCs. (**G**) Hoechst33258-stained nuclei. (**H**) Merged images. Scale bar = 200 µm.

Lv-PSPN was added to infect rMSCs with 10 MOI. After 72 h of infection, cells were observed using an Axiophot fluorescence microscope. Almost 100% of the GFP-positive rMSCs were observed (Data not shown). Western blot and RT-PCR analysis were used to verify the expression of PSPN genes after rMSCs had been transduced with Lv-PSPN (Fig. 4 A–D). Immunofluorescence was also used to show that rMSCs expressed the PSPN genes effectively (Fig. 4 E–H).

### Lv-PSPN-MSCs Protected against 6-OHDA-induced Toxicity in MES23.5 Dopaminergic Neurons

**Figure 5:**
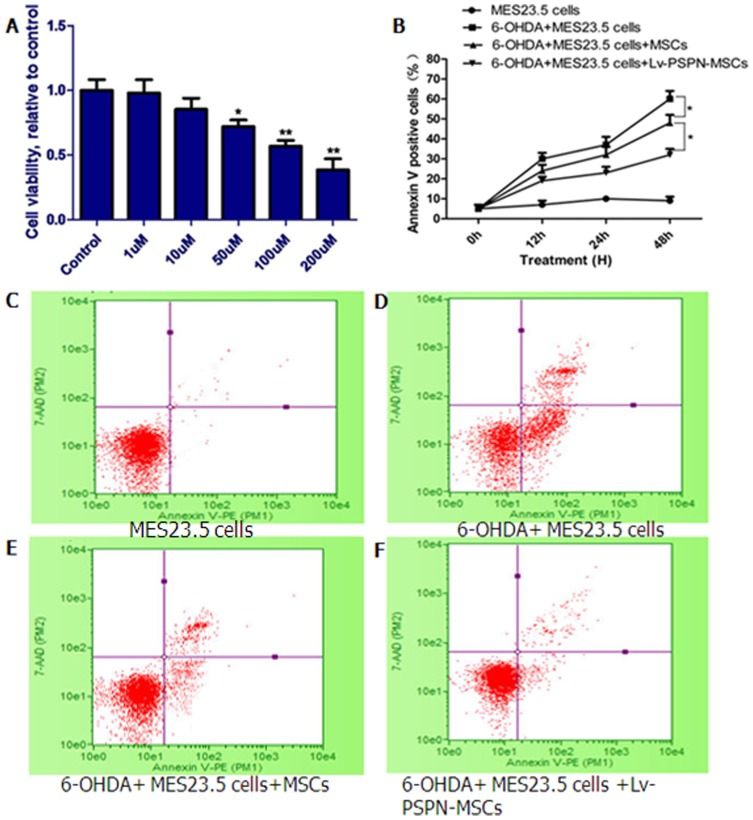
Attenuation of 6-OHDA-induced cell death by Lv-PSPN-MSC in MES23.5 cells. (A) Effect of different concentrations of 6-OHDA (1–200 µM) on metabolic rate. (B) The apoptotic rates of the MES23.5 cells treated in (C, D, E, F) were assessed at the indicated time periods by annexin V/7-AAD staining. (C) MES23.5. (D) MES23.5+6-OHDA. (E) 6-OHDA+MES23.5+MSCs. (F) 6-OHDA+MES23.5+ Lv-PSPN-MSCs. Cells were analyzed for annexin V/7-AAD staining by flow cytometry. Events in each of the four quadrants are as follows: Lower left, viable cells; lower right, cells in the early-to mid-stages of apoptosis; upper right, cells in the late-stages of apoptosis; upper left, mostly nuclear debris.*P<0.05.

Results showed exposure to Lv-null and Lv-PSPN for 72 h did not alter cell viability (Data not shown). 6-OHDA treatment induced neuronal death in a dose-dependent manner by MTS assay. Exposure to 1, 10, 50, 100, 200 µM 6-OHDA for 48 h resulted in a loss of 1%, 10%, 20%, 32%, 58% of MES23.5 cells, respectively, compared with the vehicle control (Fig. 5A). In order to observe the effect of Lv-PSPN-MSCs on cell apoptosis, we performed FACS analysis on MES23.5 cells that were co-cultured with Lv-null-MSCs or Lv-PSPN-MSCs respectively after 6-OHDA (100 µM) treatment. As shown in (Fig. 5 B–F), decreased cell apoptosis was observed in MES23.5 cells co-cultured with Lv-null-MSCs or Lv-PSPN-MSCs (compare with MES23.5 cells treated with 6-OHDA) as expected. In addition, MES23.5 cells co-cultured with Lv-PSPN-MSCs decreased cell apoptosis than that of Lv-null-MSCs, indicating the protective effect of PSPN on MES23.5 cells against 6-OHDA treatment.

### Intrastriatal Cells Transplantation

**Figure 6:**
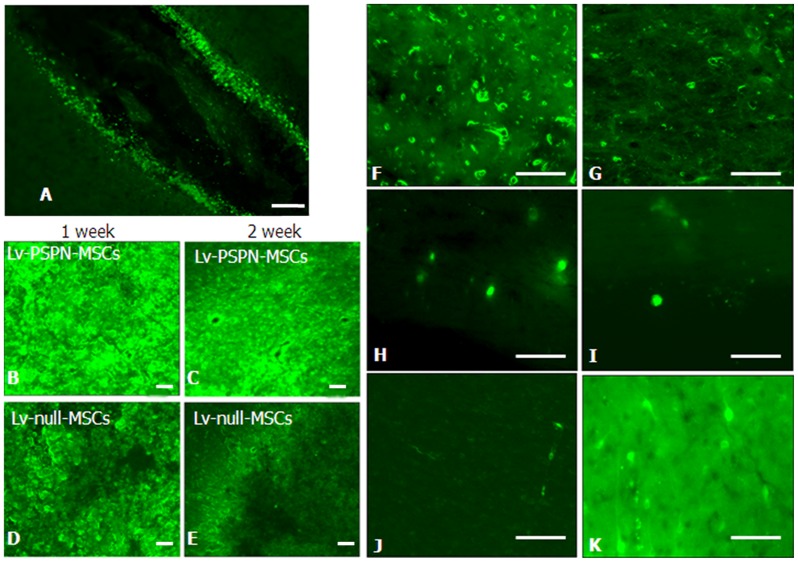
The change of EGFP-labeled rMSCs after stereotactic injection into the striatum of PD rats. (**A**) Cells were injected into the striatum through needle passage with microsyringe. (**B–E**) The fluorescence intensity in Lv-PSPN-MSCs group is stronger than Lv-null-MSCs group no matter in the first week or second week. Over a period of 4 weeks following grafting, EGFP-labeled rMSCs could be detected at various locations. rMSCs could be detected within the striatum (**F**) while others had already attached either to the contralateral striatum (**G**), periventricular regions (**H–I**) and cerebral cortex (**J**). Single cells even exhibit processes resembling neuronal morphologies (**K**). Scale bar = 200 µm.

One week and two weeks post-injection, cells could be easily identified by their green fluorescence in cryostat sections of the injection site (Fig. 6). However, the fluorescence intensity was obvious different in each group (Fig. 6B–E). PSPN over-expression improved survival rate of rMSCs. Over a period of 4 weeks following grafting, Lv-PSPN-MSCs could be detected at various locations. Lv-PSPN-MSCs could be detected within the striatum (Fig. 6 F), which was used as the target structure for the injection, while others had already attached either to the contralateral striatum (Fig. 6 G), periventricular regions (Fig. 6 H–I) and cerebral cortex (Fig. 6 J). Single cell even exhibited processes resembling neuronal morphologies (Fig. 6 K).

### Morphological investigations together with immunofluorescence

**Figure 7:**
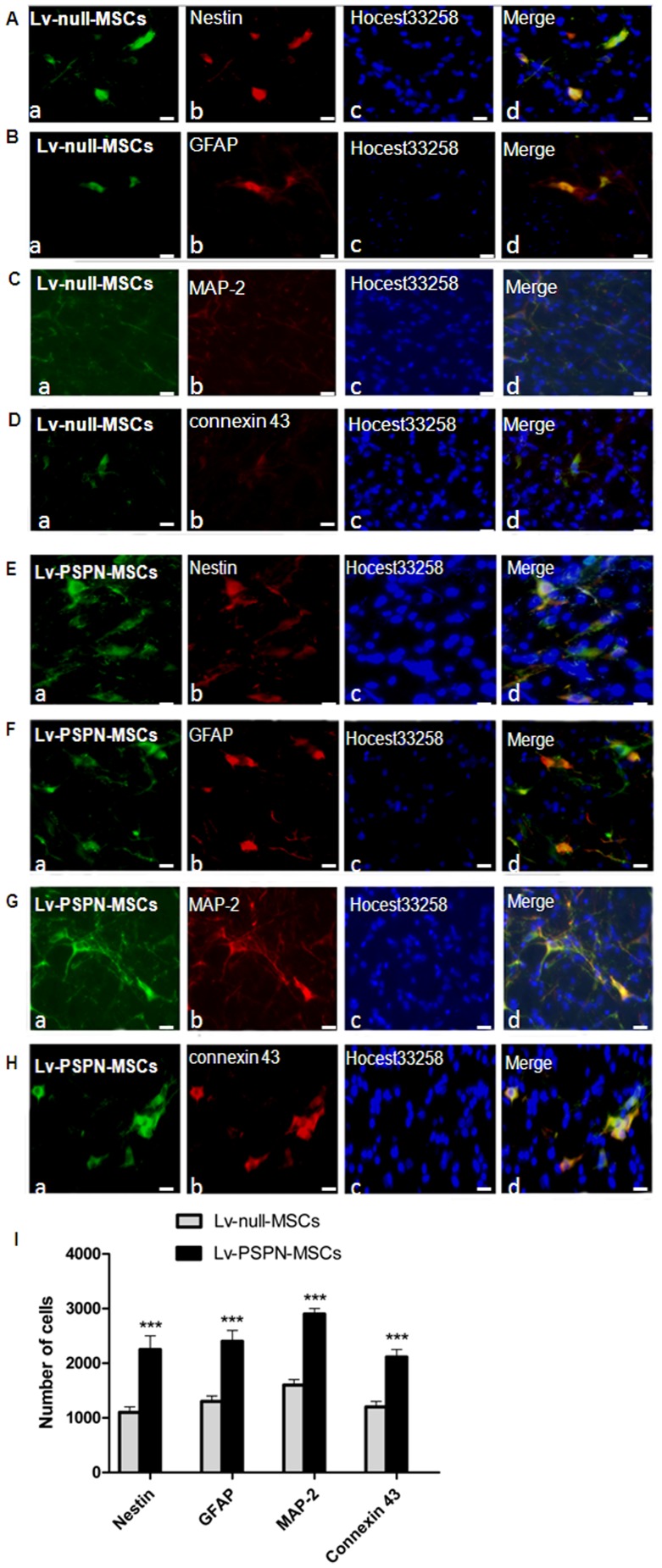
Immunofluorescence analysis of neural marker expression in EGFP-labeled rMSCs after settling in the striatum of PD rats. Fluorescence microscopic analysis of EGFP-labeled rMSCs striatal sections in Lv-null-MSCs group and Lv-PSPN-MSCs group four weeks after cell transplantation. Transplanted Lv-null-MSCs (green) were immunostained against cell markers Nestin (**A-b; red**), GFAP (**B-b; red**), MAP-2(**C-b; red**), connexin 43(**D-b; red**). Corresponding merged images are shown accordingly (**d**). Images from the Lv-PSPN-MSCs group (**E–H**) are stained with the same cell markers as the Lv-null-MSCs group. (**I**) Quantitation of Nestin, GFAP, MAP-2, connexin 43-positive cells in the striatum from Lv-null-MSCs group and Lv-PSPN-MSCs group. Error bars represent SD, ***P<0.001. Scale bar = 200 µm.

Analysis using antibodies against Nestin (Fig. 7; A-b, E-b), GFAP (Fig. 7; B-b, F-b), MAP-2 (Fig. 7; C-b, G-b) and connexin 43 (Fig. 7; D-b, H-b) revealed a morphological integration of EGFP-labeled rMSCs into the environment of host cells in the striatum. Using fluorescence microscope, a co-expression of neural marker proteins and EGFP was detectable in transplanted cells. In some cases these cells even exhibited typical neuronal or glial morphologies. The quantitative results showed a significantly higher number of Nestin, GFAP, MAP-2, connexin 43 positive cells in the Lv-PSPN-MSCs group than in the Lv-null-MSCs group (Fig. 7 I).

### Rotational Behavior Testing

**Figure 8:**
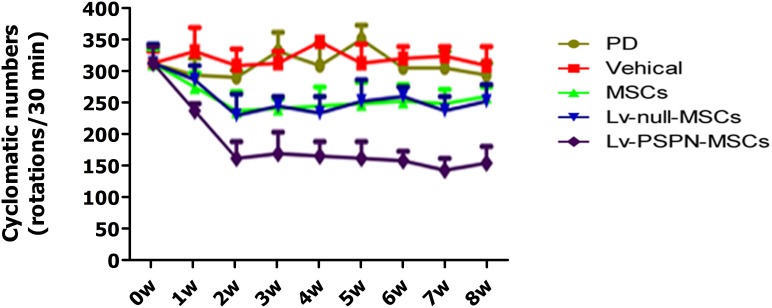
Effects of grafting with engineered rMSCs on apomorphine-induced rotational behaviors. Average rotational turns per 30 min are shown at different time points (interval = 1 week, n = 10). Data are expressed as mean ± SD. Average rotational rates of the PD group and of vehicle group were not changed from baseline. In contrast, the average rotational rates of PD rats implanted with MSCs or Lv-null-MSCs significantly decreased (*P*<0.05). The greatest decrease, however, was observed in PD rats implanted with Lv-PSPN-MSCs (*P*<0.001). In addition, this group showed significantly greater behavioral improvement than that of MSCs group (*P*<0.05) or Lv-null-MSCs group (*P*<0.05).

The apomorphine-induced rotational behaviors of all groups were shown as Fig. 8. The values are the average rotational turns per 30 minute, and the values for each week after grafting are compared to the values before grafting; in all cases, the interval between neighboring values is 1 week. Data are expressed as mean ± SEM. Average rotational rates of the PD group and of vehicle group were unchanged from the baseline. In contrast, the average rotational rates of PD rats implanted with rMSCs or Lv-null-MSCs decreased relative to the pretreatment baseline (*P*<0.05). The average rotational rates of PD rats implanted with Lv-PSPN-MSCs were lower than other groups (*P*<0.05).

### Striatal Dopamine Levels after Cell Transplantation

**Figure 9:**
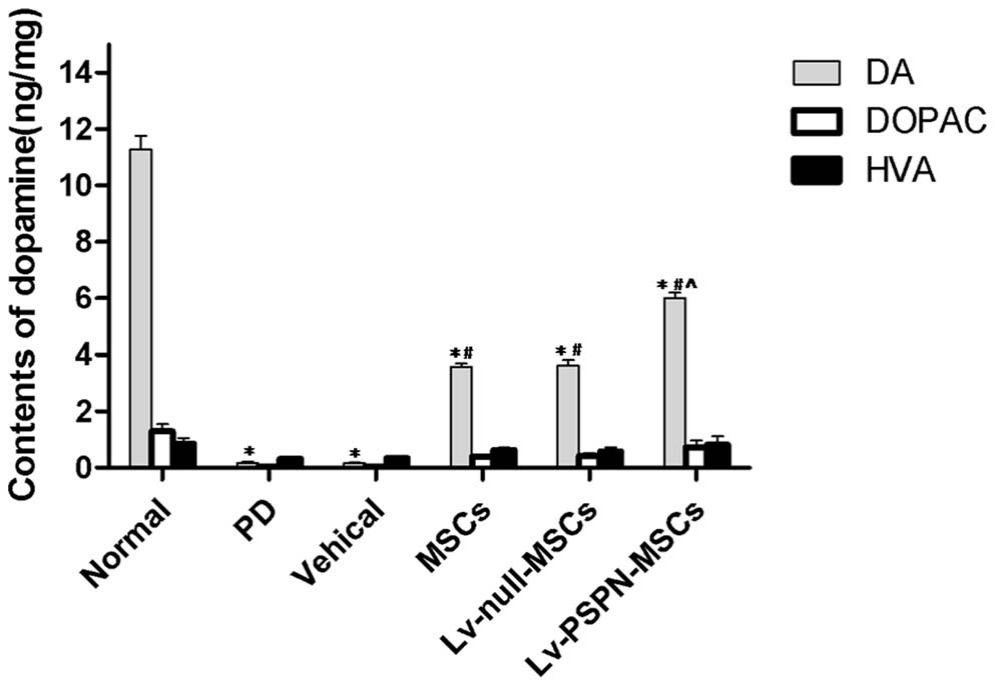
Striatal levels of DA and DOPAC were measured at 8 weeks after grafting with the different rMSCs groups. Mean DA level in the striatum of PD animals was 91.6% lower than that of control (0.53±0.08 vs. 11.28±0.65 ng/mg brain tissue). There was no significant difference between PD group and vehicle group. After transplantation with engineered rMSCs, the mean striatal DA level in the untransduced MSCs, LV-null-MSCs group and LV-PSPN-MSCs group was higher than that in PD rat, respectively. Furthermore, the DA levels in LV-PSPN-MSCs group was also greater than that in rats treated with MSCs or LV-null-MSCs. Bars indicate mean ± SD. (*P<0.05, compared to the control; ^#^ *P*<0.05, compared to the PD group; ^∧^ *P*<0.05, compared to MSCs or LV-null-MSCs group).

Dopamine levels in the striatum were determined by HPLC. Mean DA level in the striatum of PD group was lower than that of control (0.53±0.08 vs. 11.28±0.65 ng/mg brain tissue). There was no significant difference between PD group and vehical group. After transplantation with MSCs or LV-null-MSCs, DA level was increased by 186.22% (*P*<0.05) and 192.73% (*P*<0.05) compared with PD group. Furthermore, the DA concentration in the LV-PSPN-MSCs group was higher than that of PD group, MSCs group and LV-null-MSCs group (*P*<0.05, Fig. 9).

## Discussion

Adult bone marrow stromal cells (BMSC) are of significant interest in cell therapy, regarding their accessible location, low immunogenicity and multipotentiality. Previous studies already described their neural differentiation abilities [19], [20], and confirmed their potential use in the treatment of neurological diseases. Neural marker expression observed by different investigators comprises nestin and GFAP [19], nestin, NSE and neurofilament-M [20], β3-tubulin, NSE and vimentin [21], as well as myelinic basic protein (MBP), TH and also β3-tubulin [22]. Our data also provided evidence that bone marrow stroma-derived mesenchymal stem cells expressed a variety of neuronal and glial marker proteins including GFAP, Nestin, β3-tubulin, NES, s100 and IBA-1.

In addition, the neural-like character of BMSCs was supported by the potency of mesenchymal stem cells to secrete neurotrophic factors such as NGF, BDNF and GDNF [23]. These findings clearly demonstrated that even if it is not possible to direct the differentiation of a major BMSC population into true neuronal cells, they can be regarded as mini-bioreactors that are able to produce a variety of cytokines and trophic factors being important in the cure of neurodegenerative diseases. It is known that BMSCs express many cytokines which were also involved in hematopoiesis [24]. Furthermore, they supplied autocrine, paracrine and juxtacrine factors that influenced the cells of the marrow microenvironment itself [25]. Consequently, bone marrow-derived mesenchymal stem cells may be regarded as a therapeutic cell population for neurodegenerative diseases. By synthesizing neurotrophic factors in situ, they might have the potency to support the regenerative capacity of the host tissue, even if they only show in very rare cases signs of a transdifferentiation capacity. Such a therapeutic potency has already been indicated as a possible cell therapy in the rat stroke model [23]. These features make MSCs an attractive candidate to deliver therapeutic molecules to the diseased brain. However, low survival rate of MSCs when injected into the brainis still a problem.

Nowadays gene therapy raises the prospect of treatments for PD. One of gene therapy strategies for PD is to induce the generation of dopaminergic neurons from embryonic stem cells by delivering neurotrophic factors to embryonic stem cells [26]. Four members of the GDNF family of neurotrophic factors have been identified so far (GDNF, NTN, PSPN, and EVN/ART). All of them signal through binding to a specific GPI-linked GFRa receptor and in combination with a common transmembrane tyrosine kinase, cRET. GFRa-4 is the specificity-conferring coreceptor for PSPN [27], which was found to be expressed both in the ventral tegmental area, SN pars compacta (SNc) and striatum of the adult rat brain at low levels by radioactive in situ hybridization [27]. Further study confirmed midbrain dopamine neurons expressed GFRα4 [28]. In addition, it was reported that recombinant rat GFRa-4 variants could be at least partially secreted from the cells in mammalian cells, indicating soluble GFRa-4 receptor isoforms could exert its function to present PSPN to cells expressing cRET but no GFRa-4 [27]. This made it possible that PSPN could exert its protective effect though GFRα-4 mediated signaling.

Just as GDNF, PSPN has been shown to exhibit neurotrophic activity on mesencephalic dopaminergic neurons as well [29]. In vitro, treatment of dissociated neurospheres with PSPN caused a significant increase in the number of Nurr1-, Pitx3-, and TH-positive cells compared with the untreated controls [30]. The purpose of the present study points to a neurorescue activity of PSPN in the 6-OHDA-induced rat model of PD. This study established the neuroprotective effects of PSPN in experiment using both in vivo and in vitro methods. 6-OHDA is a neurotoxin classically used to produce animal models of PD. 6-OHDA-treated animals exhibit the major hallmarks of PD, including the loss of dopaminergic neurons in the SN and decrease in TH-positive neurons [31]. In this study, we observed that PSPN could restore the severe reduction in MES23.5 cell count caused by 6-OHDA treatment. This indicated that PSPN have significant protective effects against the toxic effects of 6-OHDA by rescuing MES23.5 dopaminergic neurons in vitro.

Addition of DA-producing cells into the striatum is necessary to replace DAergic neurons lost from the SN during PD, particularly during the late stages of the disease. Gene transfer provides a tool to accomplish this by genetically modifying cells in order to make them produce particular neurotransmitters [32]. So, we hypothesize that if MSCs have a relatively high expression of PSPN, it might be easier to improve survival rate of MSCs or differentiate into neuron-like cells. Therefore, we transduced target gene PSPN into MSCs effectively with lentivirus. Results showed that PSPN can stably express in vitro. An obvious therapeutic potency can be seen from our grafting experiments with the striatum as the target structure. Results showed that survival rate increased obviously in Lv-PSPN-MSCs group compared to Lv-null-MSCs group two or four weeks after transplantation. After four weeks, behavioral testing indicated that rats injected with Lv-PSPN-MSCs showed significantly lower rotational rates compared with the rats injected with Lv-null-MSCs. HPLC showed DA level in striatum of Lv-PSPN-MSCs group was significantly higher than those of other groups. Morphological investigations together with immunofluorescence analysis revealed a morphological integration of EGFP-labeled MSCs into the environment of host cells in the striatum. Using fluorescence microscope, a co-expression of neural marker proteins and EGFP was detectable in transplanted cells. For the same neural marker, the expression quantity in Lv-PSPN-MSCs group was greater than that in Lv-null-MSCs group. In some cases these cells even exhibited typical neuronal or glial morphologies. These results suggested that PSPN played an important role in improving survival rate of MSCs.

In conclusion, our data of the present study supports the standpoint that exogenous PSPN has a promising future for the clinical application of MSCs in gene therapy to treat PD. Although bone marrow stromal cells are known to express the glial as well as neuronal cell maker [23]–[26]. How to further improve maturity of MSCs compared with nerve cells no matter from the morphology of the cells or in terms of functionality remains to be determined. Moreover, further studies on MSCs need to be carried out in order to characterize mechanisms that are able to direct differentiation of MSCs into true neuronal traits. If actual morphological and functional specifications are achieved, MSCs could become a real and appropriate cell source for the use in the autologous treatment of neurological diseases.

## Supporting Information

Figure S1 **Photomicrographs of coronal sections stained for tyrosine hydroxylase (TH).** A: control, B: Lv-null-MSCs, C: Lv-PSPN-MSCs.
(TIF)

Figure S2 **The expression of persephin protein in cell conditioned medium.** Results showed that the expression of PSPN protein in conditioned medium of transduced cells was significantly increased compared to the control.
(TIF)
