# Supplementary figures and images for: The Effect of Lentivirus-Mediated PSPN Genetic Engineering Bone Marrow Mesenchymal Stem Cells on Parkinson’s Disease Rat Model
Source: PLoS One. 2014 Aug 13;9(8):e105118. doi: 10.1371/journal.pone.0105118 (PMC4132064; doi:10.1371/journal.pone.0105118)

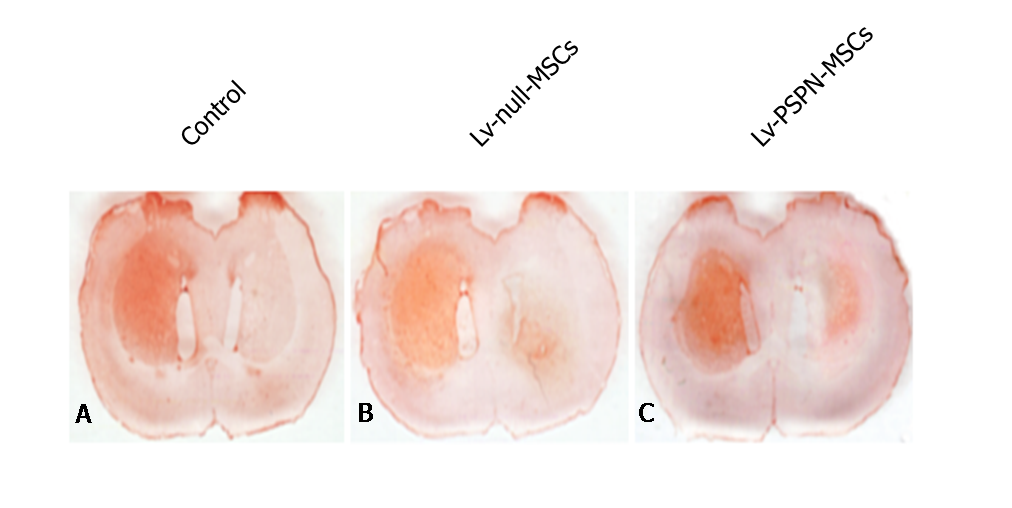

Supplement: Figure S1 — Photomicrographs of coronal sections stained for tyrosine hydroxylase (TH). A: control, B: Lv-null-MSCs, C: Lv-PSPN-MSCs. (TIF) [file pone.0105118.s001.tif]
